# Machine learning models for predicting plastic bronchitis in hospitalized children with *Mycoplasma pneumoniae* pneumonia undergoing clinically indicated bronchoscopy: a single-center retrospective study

**DOI:** 10.3389/fped.2026.1983032

**Published:** 2026-10-30

**Authors:** Shuai Yu, Yifei Li, Qing Liu, Zhengfeng Xue, Yu Gao, Yuzhi Yang, Yuanxia Li

**Affiliations:** 1 Department of Pediatric Respiratory Medicine, Affiliated Hospital of Yan'an University, Yan'an, Shaanxi, China; 2 Department of Pediatric Cardiology, West China Second University Hospital, Sichuan University, Chengdu, China

**Keywords:** children, machine learning, *Mycoplasma pneumoniae* pneumonia, plastic bronchitis, risk prediction model

## Abstract

**Objective:**

To develop and compare multiple machine learning models for predicting the risk of plastic bronchitis (PB) in children with *Mycoplasma pneumoniae* pneumonia (MPP) and to establish an interpretable early risk identification tool.

**Methods:**

A retrospective study was conducted involving 702 hospitalized children with MPP (477 non-PB and 225 PB) admitted to the Affiliated Hospital of Yan'an University from January 2023 to January 2026. Clinical characteristics, laboratory findings, and imaging data were collected. Key predictors were identified using a two-stage Lasso–Boruta approach. The dataset was randomly divided into training and test sets at a ratio of 7:3. Eight machine learning models, including CatBoost, XGBoost, LightGBM, Random Forest, Logistic Regression, Support Vector Machine, Gaussian Naive Bayes, and K-Nearest Neighbors, were developed. Hyperparameters were optimized using five-fold stratified cross-validation with grid search or random search based on the *F*1 score. Model performance was evaluated using accuracy, precision, recall, *F*1 score, ROC-AUC, PR-AUC, calibration, and decision curve analysis. SHAP analysis was applied for model interpretation. Temporal validation and bootstrap optimism correction were further performed to assess temporal generalizability and optimism in model performance.

**Results:**

The Lasso–Boruta approach identified nine PB-related predictors, including fever duration, peak temperature, WBC, CRP, ALB, LDH, FDP, D-dimer, and pulmonary consolidation. All eight models demonstrated predictive capability in the test set, with ROC-AUC values ranging from 0.854 to 0.881. RF achieved an accuracy of 0.853, precision of 0.804, recall of 0.721, *F*1 score of 0.759, and ROC-AUC of 0.881, whereas CatBoost achieved an accuracy of 0.853, precision of 0.793, recall of 0.736, *F*1 score of 0.762, and ROC-AUC of 0.871. In temporal validation, RF achieved the highest accuracy (0.925) and *F*1 score (0.900), whereas CatBoost achieved the highest ROC-AUC (0.970) and PR-AUC (0.956). Considering the complementary performance profiles across different evaluation dimensions and the results of bootstrap optimism correction, RF and CatBoost were retained as candidate models for further interpretation and risk prediction tool development. SHAP analysis provided individual-level explanations of the contributions of the selected predictors to PB risk.

**Conclusions:**

Machine learning models based on clinical manifestations, laboratory indicators, and imaging features showed potential for PB risk identification in children with MPP. RF and CatBoost exhibited complementary predictive strengths across different evaluation dimensions, while SHAP analysis facilitated individual-level interpretation. Further multicenter external validation is required.

## Graphical Abstract

**Figure:**
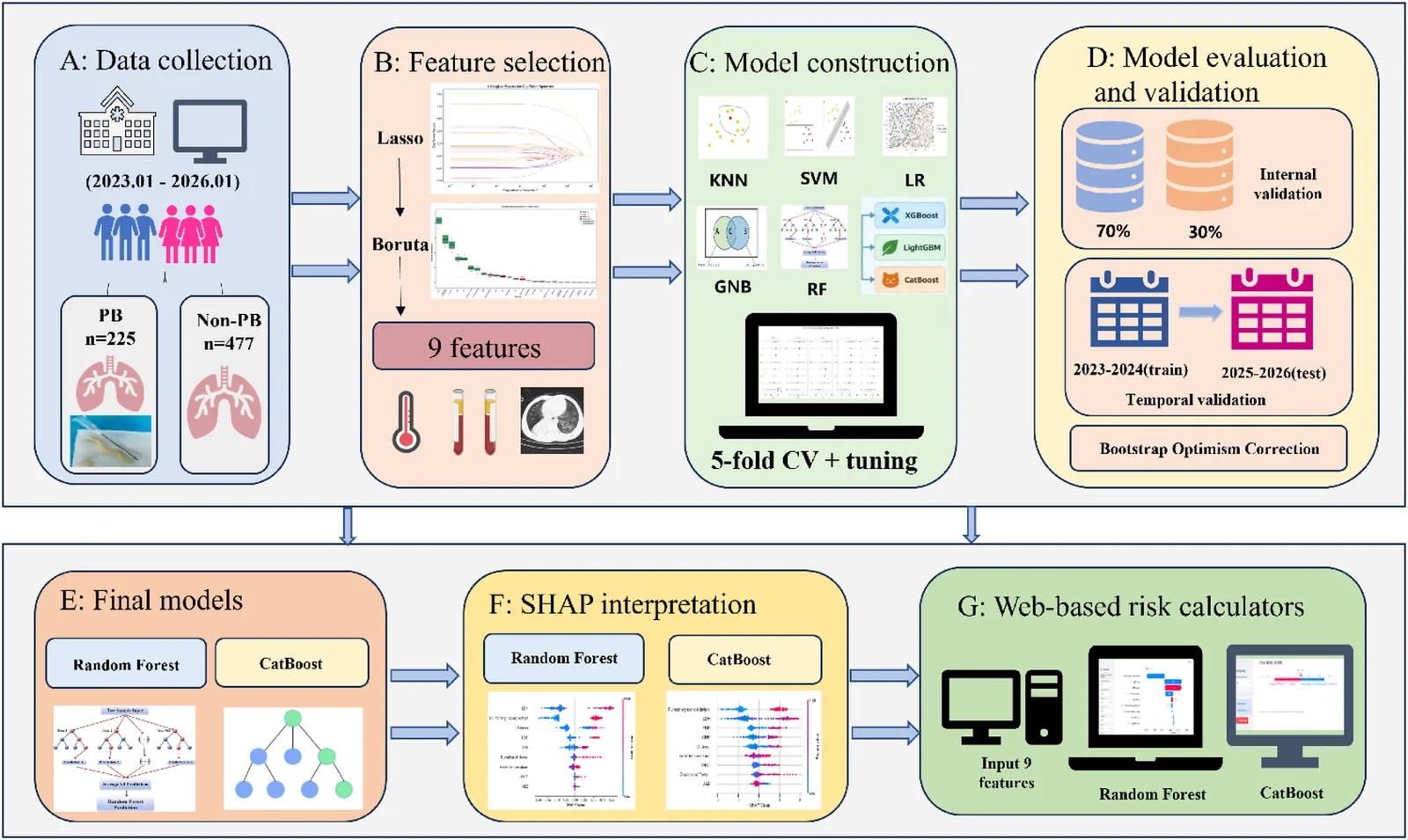

## 1 Introduction

*Mycoplasma pneumoniae* (MP) is a common pathogen causing community-acquired pneumonia in children. In recent years, the incidence of M. pneumoniae pneumonia (MPP) has remained at a relatively high level, with an increasing proportion of severe and refractory cases, posing a serious threat to pediatric health (1–4). Plastic bronchitis (PB) is a rare complication of MPP and is characterized by the formation of endogenous, branching bronchial casts within the airways, resulting in partial or complete airway obstruction. Clinically, PB commonly presents with persistent high fever, severe cough, progressive dyspnea, and atelectasis. Once bronchial casts form, progressive airway obstruction may occur, and severe cases can further develop hypoxemia, respiratory failure, or even death. Therefore, timely identification of PB and early bronchoscopic intervention are important for relieving airway obstruction and improving clinical outcomes. If the casts are not promptly removed by bronchoscopy, the disease may rapidly progress to acute respiratory distress syndrome, necrotizing pneumonia, bronchiolitis obliterans, or even death, thereby seriously affecting long-term prognosis (5–7). Because PB often has an insidious onset and rapid progression, and its definitive diagnosis relies on invasive bronchoscopy, early and noninvasive identification of PB risk in children with MPP remains an important clinical challenge. Such risk assessment may facilitate the timely identification of patients at high risk of PB before bronchoscopic confirmation and provide a basis for further clinical evaluation and intervention.

With the advancement of medical informatization, clinical data are growing rapidly. Traditional statistical methods are mostly based on assumptions of linear relationships among variables, which are insufficient for capturing the complex, nonlinear interactions between clinical indicators, thereby limiting the predictive performance and generalizability of the models (8, 9). Machine learning (ML), on the other hand, leverages advanced algorithms and statistical techniques to process data and predict disease progression, adverse outcomes, and treatment responses (10–12), and can model nonlinear relationships, complex interactions, and patterns within high-dimensional datasets. In recent years, several studies have explored risk prediction for MPP-associated PB in children. Previous studies have developed nomograms based on logistic regression and have further introduced machine-learning approaches for PB risk prediction. For example, XGBoost, logistic regression, random forest (RF), and support vector machine (SVM) have been used to construct PB prediction models, with LASSO and Boruta applied for feature selection and SHAP used for model interpretation; web-based risk prediction tools have also been developed (13). In addition, other studies have integrated clinical variables with chest CT radiomic features to construct machine-learning models for identifying PB before bronchoscopy (14). These studies suggest that machine learning has potential value for identifying the risk of MPP-associated PB. However, existing studies have mainly focused on comparisons among a limited number of algorithms or on models based on specific imaging features, and there remains room for further investigation through systematic comparison of different machine-learning algorithms, assessment of model robustness, and individualized prediction interpretation. Therefore, the present study focused on a specific population of hospitalized children with MPP who underwent bronchoscopy for clinical indications. Clinical characteristics, laboratory findings, and imaging variables were systematically collected, and key predictors were selected using a two-stage Lasso–Boruta procedure. Multiple machine-learning algorithms were then compared in terms of predictive performance. On this basis, the final candidate models were further evaluated using multidimensional performance metrics, temporal validation, and SHAP-based interpretability analysis, and corresponding individualized risk prediction calculators were developed. The aim was to provide a scientific basis for the early identification of children at high risk of PB in this population, timely intervention, and optimization of clinical decision-making.

## 2 Materials and methods

### 2.1 Study population

This study was a single-center retrospective case analysis. We consecutively screened 750 hospitalized children with *Mycoplasma pneumoniae* pneumonia (MPP) who were admitted to the Affiliated Hospital of Yan'an University between January 2023 and January 2026. After excluding 48 patients who did not meet the eligibility criteria, a total of 702 hospitalized children who underwent fiberoptic bronchoscopy examination and treatment for clinical indications were included in the final analysis. The patient selection process is shown in Figure 1. The study protocol was approved by the Ethics Committee of the Affiliated Hospital of Yan’an University (IIT-R-2026207). The study was conducted in accordance with the Declaration of Helsinki and current medical ethical standards, with strict protection of patient privacy and appropriate data security measures. As this was a retrospective study, all data were extracted from the hospital medical record system and anonymized, with no personally identifiable information included. The Ethics Committee approved a waiver of written informed consent from the patients' parents or legal guardians.

**Figure 1:**
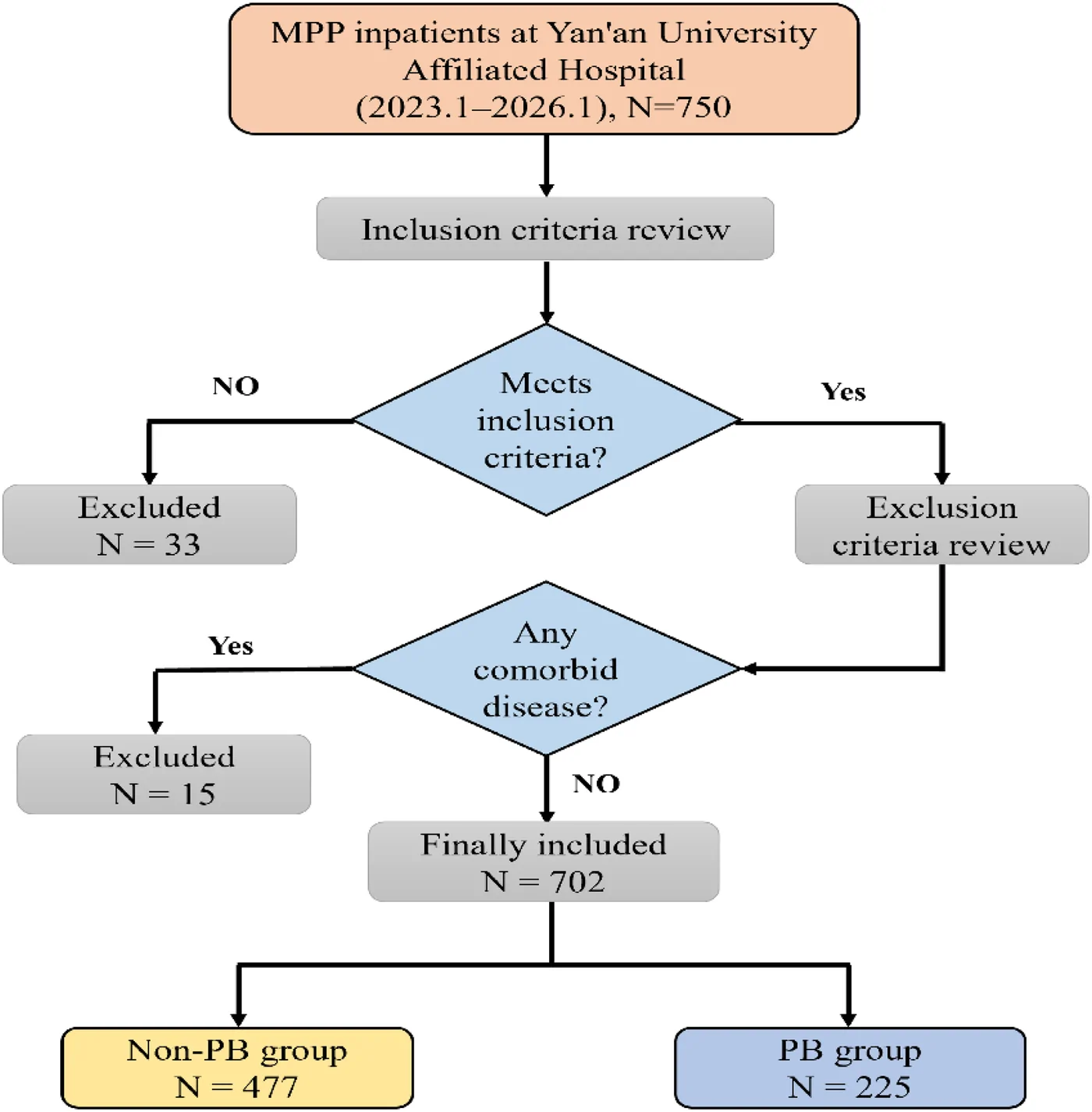
Study participant flow diagram.

### 2.2 Methods

#### 2.2.1 Diagnostic criteria for MPP and PB

The diagnostic criteria for MPP were based on the Guidelines for the Diagnosis and Treatment of *Mycoplasma pneumoniae* Pneumonia in Children (2023 Edition) (15). The diagnosis of PB was confirmed when a cast specimen with a bronchial branching shape was extracted via flexible bronchoscopy (16). All bronchoscopic procedures were performed in accordance with the Chinese Pediatric Flexible Bronchoscopy Guidelines (2018 Edition) (17).

#### 2.2.2 Inclusion and exclusion criteria

Inclusion criteria: ① age ≤ 14 years; ② meeting the diagnostic criteria for MPP according to the Guidelines for the Diagnosis and Treatment of *Mycoplasma pneumoniae* Pneumonia in Children (2023 Edition) (15); ③ having indications for bronchoscopy without contraindications, as per the Chinese Pediatric Flexible Bronchoscopy Guidelines (2018 Edition) (17); ④ for PB patients, bronchial cast specimens with branching shapes were retrieved during bronchoscopy.

Exclusion criteria: complicated with underlying diseases such as tuberculosis, bronchial asthma, congenital heart disease, neuromuscular disorders, immunodeficiencies, or hereditary/metabolic diseases.

#### 2.2.3 Study grouping

Patients were divided into a non-PB group and a PB group based on whether bronchial cast specimens with branching shapes were retrieved during bronchoscopy.

#### 2.2.4 Clinical data collection

The following clinical data were collected for each patient: age, sex, single infection, mixed infection, duration of fever, peak temperature, wheezing, tachypnea, dyspnea, wheezing sounds, abdominal pain, vomiting, hepatic impairment, myocardial impairment, white blood cell count (WBC), neutrophil percentage (NEU), lymphocyte percentage (LYM), red blood cell count (RBC), hemoglobin (HB), platelet count (PLT), C-reactive protein (CRP), albumin (ALB), lactate dehydrogenase (LDH), fibrinogen (FIB), fibrinogen degradation products (FDP), plasma D-dimer, pulmonary consolidation, atelectasis, and pleural effusion. Hospital admission was defined as the prediction time origin (time zero). The models were designed to predict the risk of plastic bronchitis (PB), as confirmed by fiberoptic bronchoscopy, using clinical information, laboratory parameters, and imaging findings available early after admission. All predictor variables were obtained at admission or from clinical assessments, laboratory tests, and imaging examinations performed within 24 h after admission, and all were collected before fiberoptic bronchoscopy and confirmation of PB. Fever duration and peak body temperature were recorded based on the medical history obtained at admission rather than on clinical-course data collected after bronchoscopy.

All children underwent non-contrast chest computed tomography (CT) within 24 h after admission. The imaging findings were independently reviewed by two experienced radiologists. In cases of disagreement, the final interpretation was determined through discussion with a third senior radiologist. All imaging examinations were completed before fiberoptic bronchoscopy and the diagnosis of PB. All blood samples were collected within 24 h after admission.

#### 2.2.5 Missing data handling

During retrospective data collection, some clinical variables had missing values. Directly excluding cases with missing values could reduce the sample size and introduce selection bias. Therefore, the proportion of missing data for each variable was first evaluated. Variables with missing rates exceeding 20% were designated for exclusion, whereas those with missing rates of 20% or less were handled using a random forest-based iterative imputation approach implemented with IterativeImputer and RandomForestRegressor. Specifically, random forest regression models were iteratively fitted to estimate missing values based on the observed information from other variables. This approach can capture complex nonlinear relationships and interactions among variables without requiring predefined parametric distributions, thereby reducing information loss.

During the development of the prediction models, the dataset was first divided into training and testing sets. The imputation model was fitted exclusively on the training set and subsequently applied to the testing set without refitting. This procedure prevented the testing data from contributing to the fitting of the imputation model, thereby minimizing the risk of data leakage during model evaluation.

#### 2.2.6 Feature selection, model development, and internal validation

A two-stage Lasso–Boruta approach was applied for feature selection. First, the 702 patients were split into a training cohort (*n* = 491) and a testing cohort (*n* = 211) at a ratio of 7:3 using stratified random sampling based on the outcome variable, with a fixed random seed (42) to ensure reproducibility. All preprocessing and feature selection procedures were performed exclusively within the training cohort to prevent data leakage. Continuous variables were standardized using *Z*-score normalization, with scaling parameters estimated from the training cohort and subsequently applied to both training and testing cohorts. L1-regularized logistic regression (Lasso logistic regression) was initially used for preliminary feature reduction. The optimal regularization parameter C was determined by minimizing log loss through 10-fold cross-validation, and features with non-zero coefficients were retained. The selected features were subsequently entered into the Boruta algorithm for further selection. Boruta was independently performed for 10 iterations. In each iteration, the maximum importance of the shadow features was used as the selection threshold, and real features with importance values exceeding this threshold were recorded as “hits.” The statistical significance of feature importance was assessed based on a binomial distribution test to determine whether each feature was significantly more important than random noise. Only features classified as “confirmed” in at least five Boruta runs were retained as the final predictor set.

Using PB vs. non-PB classification as the prediction target, eight machine learning models were developed, including Categorical Boosting (CatBoost), Extreme Gradient Boosting (XGBoost), Light Gradient Boosting Machine (LightGBM), Random Forest (RF), Logistic Regression (LR), Support Vector Machine (SVM), Gaussian Naive Bayes (GNB), and K-Nearest Neighbors (KNN). For XGBoost, LightGBM, RF, LR, SVM, GNB, and KNN models, categorical variables were transformed using one-hot encoding. For the CatBoost model, categorical variables were retained in their original format to utilize its native categorical feature handling capability. Continuous variables were standardized using *Z*-score normalization, with scaling parameters estimated only from the training cohort and applied to the testing cohort. During model training, hyperparameter optimization was performed within the training cohort using 5-fold cross-validation combined with grid search or random search. *F*1 score was used as the criterion for hyperparameter selection, and the optimal parameter combination for each model was determined. The final models were subsequently retrained using the complete training cohort and evaluated in the independent testing cohort. Model performance was assessed using Accuracy, Precision, Recall, *F*1 score, and ROC-AUC. ROC curves, precision–recall curves, calibration curves, and decision curve analysis (DCA) were generated to comprehensively evaluate model discrimination, classification performance, probability calibration, and clinical utility. Based on these multidimensional evaluations, candidate models for subsequent model interpretation and risk prediction tool development were selected. To assess potential overfitting, performance differences between the training and testing cohorts were compared. The absolute differences (performance gaps) between the training and testing sets were calculated for ROC-AUC, Accuracy, Precision, Recall, and *F1* score. Dataset splitting was performed based on unique patient identifiers to ensure that the same patient did not appear in both training and testing cohorts. In addition, learning curves were generated to evaluate changes in model performance with increasing training sample size and to assess model stability.

#### 2.2.7 Temporal validation and bootstrap optimism correction

To further evaluate the temporal generalizability of the models, temporal validation was performed. According to the admission date, patients admitted between January 2023 and December 2024 (*n* = 622) were assigned to the model development cohort, whereas those admitted between January 2025 and January 2026 (*n* = 80) were assigned to the temporal validation cohort. In addition, bootstrap optimism correction was applied to assess potential optimism bias in model performance.

#### 2.2.8 Sensitivity analysis

To assess the potential influence of time-related variables, including fever duration and peak temperature, on model performance, sensitivity analyses were performed. After excluding fever duration and peak temperature, the prediction models were reconstructed using the same preprocessing, feature selection, model training, and evaluation procedures as the primary analysis. The changes in model performance metrics, including ROC-AUC, Accuracy, Recall, and *F*1 score, were compared between the sensitivity analysis and the primary analysis to evaluate the robustness of the findings.

#### 2.2.9 Model interpretation

SHAP (Shapley Additive Explanations) was applied to interpret the final retained candidate models. The contribution of each predictor variable to model output was quantified to evaluate feature importance and the direction of its influence on individual risk predictions. SHAP summary plots and related visualizations were generated to illustrate overall feature contributions and patterns of predictor effects.

#### 2.2.10 Risk prediction calculator development

Based on the final retained RF and CatBoost models, two individualized risk prediction calculators were developed. The calculators incorporated the predictors included in the corresponding final models and were designed to accept patient-level clinical, laboratory, and imaging information available within 24 h after admission. For each patient, the fitted model generated an individualized predicted probability of PB. The calculators were developed to facilitate convenient application of the prediction models and provide an interpretable estimate of individual PB risk before bronchoscopic confirmation. Both calculators were implemented using Streamlit as separate model-specific pages within a single web application and deployed on Streamlit Community Cloud for public access.

### 2.3 Statistical analysis

Statistical analyses were performed using SPSS 26.0. Normally distributed continuous data were expressed as mean ± standard deviation $\left(\bar{x}\pm s\right)$; comparisons between groups were made using the *t*-test for equal variances and the *t*’-test for unequal variances. Non-normally distributed continuous data were expressed as median (interquartile range) [*M* (Q1, Q3)] and compared using the Wilcoxon rank sum test. Categorical variables were presented as frequencies and percentages [*n* (%)], and group comparisons were performed using the Pearson chi-square test; when the expected frequency in ≥20% of cells was <5, Fisher's exact test was used instead. Machine learning modeling and SHAP visualization were conducted using Python 3.9.6 and Jupyter Notebook, with libraries including numpy, pandas, matplotlib, seaborn, and lightgbm.

## 3 Results

### 3.1 Comparison of clinical data between non-PB and PB groups

A total of 702 children with MPP were enrolled in this study, including 477 in the non-PB group and 225 in the PB group. There were no statistically significant differences in age or sex between the two groups (*P* > 0.05). No significant between-group difference was observed in seasonal distribution. Compared with the non-PB group, children in the PB group had longer fever duration and higher peak fever, as well as higher incidences of wheezing, tachypnea, abdominal pain, and vomiting (*P* < 0.05). In terms of laboratory findings, the PB group showed elevated levels of WBC, NEU%, CRP, LDH, FIB, FDP, and D-dimer, while LYM%, RBC, HB, and ALB levels were decreased (*P* < 0.001). Regarding imaging manifestations, the PB group had higher incidences of pulmonary consolidation (88.44% vs. 29.14%), atelectasis (16.00% vs. 1.47%), and pleural effusion (31.56% vs. 5.24%) compared with the non-PB group (*P* < 0.001); see Table 1.

**Table 1:** Comparison of clinical data between the non-PB and PB groups [*n* (%), median (Q1, Q3)].

Variable	Non-PB (*n* = 477)	PB (*n* = 225)	Statistic (*χ*^2^/*Z*)	*P* value
Age	7 (5, 9)	7 (5, 9)	−0.553	0.580
Sex			1.534	0.215
Male	239 (50.10)	124 (55.11)		
Female	238 (49.90)	101 (44.89)		
Infection			1.392	0.238
Single infection	94 (19.71)	36 (16.00)		
Mixed infection	383 (80.29)	189 (84.00)		
Season			2.527	0.470
Spring	46 (9.64)	18 (8.00)		
Summer	56 (11.74)	27 (12.00)		
Autumn	232 (48.64)	100 (44.44)		
Winter	143 (29.98)	80 (35.56)		
Duration of fever	5 (2, 6.5)	7 (5, 10)	−10.83	<0.001
Peak temperature	39 (38.4, 39.5)	39.5 (39, 40)	−9.162	<0.001
Wheezing	17 (3.56)	16 (7.11)	4.294	0.038
Tachypnea	8 (1.68)	14 (6.22)	10.404	0.001
Dyspnea	5 (1.05)	6 (2.67)	/	0.116
Abdominal pain	35 (7.34)	27 (12.00)	4.128	0.042
Vomiting	50 (10.48)	44 (19.56)	10.853	0.001
Hepatic impairment	7 (1.47)	8 (3.56)	/	0.093
Myocardial impairment	23 (4.82)	16 (7.11)	1.527	0.217
Wheezing sounds	19 (3.98)	11 (4.89)	0.307	0.580
WBC	7.53 (6.00, 9.56)	8.29 (6.45, 11.53)	−3.487	<0.001
NEU%	60.60 (52.40, 69.40)	65.70 (57.20, 75.65)	−4.672	<0.001
LYM%	28.25 (20.42, 36.00)	23.60 (16.55, 30.30)	−5.17	<0.001
RBC	4.59 (4.34, 4.86)	4.46 (4.10, 4.74)	−4.874	<0.001
HB	127 (121, 134)	124 (117, 131)	−3.753	<0.001
PLT	289 (241.5, 358)	292 (238, 377.5)	−0.701	0.483
CRP	9.59 (5.41, 18.11)	26.99 (11.87, 43.76)	−10.663	<0.001
ALB	41.2 (38.9, 43.45)	39.4 (36.5, 42.45)	−5.301	<0.001
LDH	279 (239, 332)	471 (371.5, 600)	−15.149	<0.001
FIB	4.3 (3.72, 4.88)	4.56 (4.02, 5.06)	−3.577	<0.001
FDP	2.5 (2.5, 2.8)	4.1 (2.5, 6.81)	−12.393	<0.001
D-dimer	0.39 (0.23, 0.71)	1.38 (0.76, 3.06)	−13.393	<0.001
Pulmonary consolidation	139 (29.14)	199 (88.44)	215.371	<0.001
Atelectasis	7 (1.47)	36 (16.00)	56.152	<0.001
Pleural effusion	25 (5.24)	71 (31.56)	89.677	<0.001

### 3.2 Identification of predictive features for PB using the Lasso–Boruta approach

To identify robust predictors associated with PB, a two-stage feature selection strategy combining Lasso regression and Boruta analysis was performed. Initially, L1-regularized logistic regression reduced the original feature set by retaining variables with non-zero coefficients. Twenty-two candidate variables were retained after Lasso selection. These variables were subsequently entered into repeated Boruta analysis to further evaluate their importance relative to randomly generated shadow features. After 10 independent Boruta iterations, nine variables were consistently classified as “confirmed” and were therefore selected as the final predictor set for model development. These variables included fever duration, peak temperature, WBC, CRP, ALB, LDH, FDP, D-dimer, and pulmonary consolidation (Figure 2).

**Figure 2:**
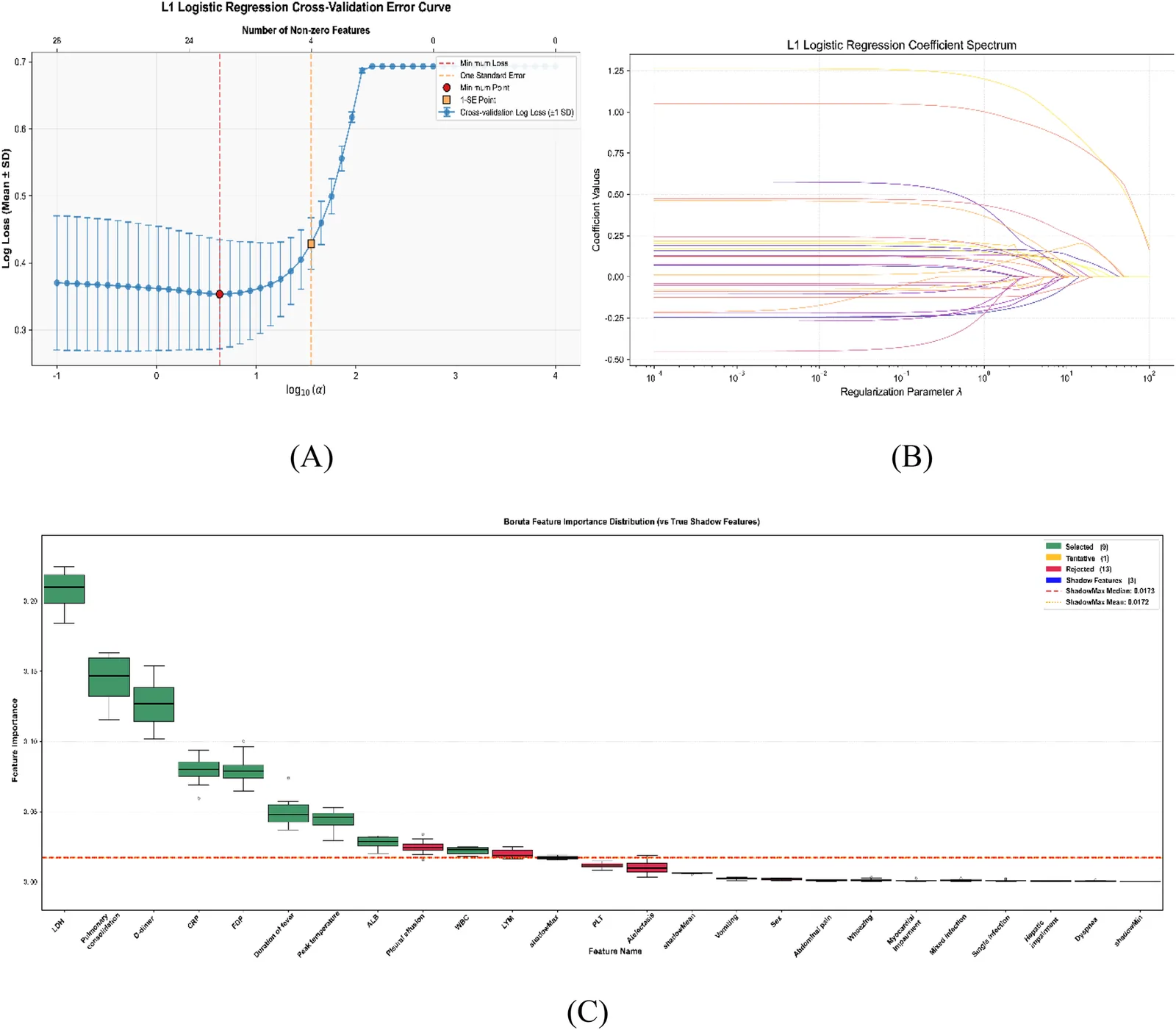
Lasso–Boruta two-stage screening plot. **(A)** Cross-validation error curves of L1-regularized logistic regression with 10-fold cross-validation; **(B)** coefficient shrinkage paths of L1-regularized logistic regression; **(C)** box plots comparing the importance score distributions of Boruta-selected features with those of shadow features.

### 3.3 Model performance evaluation

#### 3.3.1 Hyperparameter optimization and cross-validation performance of the eight models

In the training set, five-fold stratified cross-validation was performed, with the *F*1 score used as the optimization criterion for hyperparameter tuning of the eight machine learning models. The hyperparameter search spaces and the finally determined optimal hyperparameters for each model are presented in Supplementary Table S1. Based on the optimal hyperparameters determined for each model, five-fold stratified cross-validation was further performed to evaluate Accuracy, Precision, Recall, *F*1 score, and ROC-AUC. Cross-validation performance was summarized as the mean ± standard deviation and the actual range across the five folds, with the results presented in Supplementary Table S2. In addition, a forest plot was used to visualize and compare the mean performance and 95% CIs of the eight models across the five-fold cross-validation (Supplementary Figure S1).

#### 3.3.2 Overall performance and overfitting evaluation of the eight models in the training and test sets

The overall performance of the eight models in the training and test sets is presented in Table 2. In the training set, the Accuracy, Precision, Recall, *F*1 score, and ROC-AUC of KNN, XGBoost, and LightGBM all reached 1.000. For CatBoost, the corresponding values were 0.994, 1.000, 0.981, 0.990, and 1.000, respectively. The training-set ROC-AUC values of RF, LR, SVM, and GNB were 0.956, 0.923, 0.915, and 0.902, respectively. In the independent test set, the overall performance of all models decreased compared with that in the training set. The Accuracy ranged from 0.807 to 0.853, the *F*1 score from 0.667 to 0.762, and the ROC-AUC from 0.854 to 0.881. CatBoost and RF both achieved an Accuracy of 0.853, with *F*1 scores of 0.762 and 0.759, respectively; the ROC-AUC was 0.881 for RF and 0.871 for CatBoost. The ROC-AUC values of XGBoost, LightGBM, and LR were 0.878, 0.880, and 0.877, respectively, while those of SVM, KNN, and GNB were 0.875, 0.855, and 0.854, respectively.

**Table 2:** Comparison of the overall performance of the eight models in the training and test sets.

Model	Accuracy	Precision	Recall	*F*1 score	ROC-AUC
Training set					
KNN	**1.000** **(1.000–1.000)**	**1.000** **(1.000–1.000)**	**1.000** **(1.000–1.000)**	**1.000** **(1.000–1.000)**	**1.000** **(1.000–1.000)**
GNB	0.853 (0.821–0.884)	0.840 (0.775–0.897)	0.667 (0.592–0.742)	0.743 (0.683–0.797)	0.902 (0.869–0.931)
LR	0.891 (0.862–0.919)	0.856 (0.795–0.910)	0.795 (0.730–0.852)	0.824 (0.772–0.866)	0.923 (0.893–0.947)
SVM	0.886 (0.857–0.912)	0.843 (0.782–0.898)	0.789 (0.723–0.852)	0.815 (0.765–0.859)	0.915 (0.884–0.943)
XGBoost	**1.000** **(1.000–1.000)**	**1.000** **(1.000–1.000)**	**1.000** **(1.000–1.000)**	**1.000** **(1.000–1.000)**	**1.000** **(1.000–1.000)**
CatBoost	0.994 (0.986–1.000)	**1.000** **(1.000–1.000)**	0.981 (0.956–1.000)	0.990 (0.978–1.000)	**1.000** **(1.000–1.000)**
LightGBM	**1.000** **(1.000–1.000)**	**1.000** **(1.000–1.000)**	**1.000** **(1.000–1.000)**	**1.000** **(1.000–1.000)**	**1.000** **(1.000–1.000)**
RF	0.900 (0.876–0.927)	0.881 (0.826–0.929)	0.796 (0.735–0.858)	0.836 (0.791–0.878)	0.956 (0.937–0.973)
Test set					
KNN	0.820 (0.768–0.872)	0.743 (0.621–0.849)	0.677 (0.562–0.785)	0.707 (0.605–0.791)	0.855 (0.797–0.912)
GNB	0.807 (0.753–0.858)	0.747 (0.627–0.863)	0.606 (0.477–0.716)	0.667 (0.558–0.756)	0.854 (0.797–0.908)
LR	0.830 (0.777–0.877)	0.767 (0.655–0.869)	0.678 (0.562–0.788)	0.718 (0.623–0.797)	0.877 (0.823–0.927)
SVM	0.831 (0.777–0.882)	0.760 (0.650–0.860)	0.693 (0.575–0.800)	0.723 (0.624–0.803)	0.875 (0.821–0.923)
XGBoost	0.844 (0.796–0.891)	0.761 (0.655–0.859)	**0.750** **(0.648–0.849)**	0.754 (0.667–0.833)	0.878 (0.827–0.926)
CatBoost	**0.853** **(0.806–0.900)**	0.793 (0.690–0.893)	0.736 (0.630–0.833)	**0.762** **(0.678–0.838)**	0.871 (0.814–0.925)
LightGBM	0.843 (0.791–0.891)	0.768 (0.661–0.873)	0.736 (0.632–0.836)	0.750 (0.658–0.831)	0.880 (0.825–0.931)
RF	**0.853** **(0.806–0.900)**	**0.804** **(0.696–0.905)**	0.721 (0.607–0.821)	0.759 (0.672–0.836)	**0.881** **(0.832–0.930)**

Note: Bold values indicate the best results for each performance metric within the training and test sets, respectively, with the corresponding intervals also bolded.

To further evaluate the potential risk of overfitting, the performance differences between the training and test sets for each model were compared, as shown in Table 3. All models showed positive point estimates for the training–test performance differences, although the 95% confidence intervals for some metrics included zero. The largest Δ*F*1 was observed for KNN (0.293), followed by LightGBM (0.250), XGBoost (0.246), and CatBoost (0.228), while the Δ*F*1 for RF was 0.077. For ROC-AUC, the training–test differences for KNN, CatBoost, XGBoost, LightGBM, and RF were 0.145, 0.129, 0.122, 0.120, and 0.075, respectively. These results indicate that the magnitude of performance differences between the training and test sets varied across the models.

**Table 3:** Performance differences between the training and test sets for the eight models.

Model	ΔAccuracy (95% CI)	ΔPrecision (95% CI)	ΔRecall (95% CI)	Δ*F*1 (95% CI)	ΔROC-AUC (95% CI)
KNN	0.180 (0.1280 to 0.2322)	0.257 (0.1475 to 0.3667)	0.323 (0.2143 to 0.4366)	0.293 (0.2069 to 0.3818)	0.145 (0.0905 to 0.2062)
GNB	**0.046** **(−0.0119 to 0.1114)**	0.093 (−0.0371 to 0.2342)	**0.061** **(−0.0706 to 0.1969)**	**0.076 (−0.0314 to 0.1954)**	0.048 (−0.0135 to 0.1135)
LR	0.061 (0.0071 to 0.1202)	0.089 (−0.0298 to 0.2075)	0.117 (−0.0052 to 0.2494)	0.106 (0.0098 to 0.2073)	0.046 (−0.0118 to 0.1042)
SVM	0.055 (−0.0003 to 0.1135)	0.083 (−0.0329 to 0.2034)	0.096 (−0.0247 to 0.2306)	0.092 (−0.0035 to 0.1975)	**0.040** **(−0.0179 to 0.1021)**
XGBoost	0.156 (0.1090 to 0.2038)	0.239 (0.1379 to 0.3378)	0.250 (0.1493 to 0.3529)	0.246 (0.1692 to 0.3280)	0.122 (0.0738 to 0.1780)
CatBoost	0.141 (0.0934 to 0.1902)	0.207 (0.1091 to 0.3016)	0.245 (0.1438 to 0.3521)	0.228 (0.1489 to 0.3134)	0.129 (0.0751 to 0.1895)
LightGBM	0.157 (0.1090 to 0.2038)	0.232 (0.1290 to 0.3333)	0.264 (0.1636 to 0.3699)	0.250 (0.1729 to 0.3333)	0.120 (0.0703 to 0.1744)
RF	0.047 (−0.0071 to 0.1014)	**0.077** **(−0.0361 to 0.1912)**	0.075 (−0.0457 to 0.1971)	0.077 (−0.0141 to 0.1783)	0.075 (0.0222 to 0.1317)

Note: Bold values indicate the smallest absolute point estimates of the training-test performance differences for each metric; the corresponding 95% confidence intervals are also bolded.

To further evaluate the stability of the models across different training sample sizes and their potential risk of overfitting, learning curves were generated for all models using ROC-AUC as the evaluation metric to examine changes in model performance on the training and cross-validation sets as the training sample size increased (Figure 3). Overall, the ROC-AUC of the validation sets tended to stabilize as the training sample size increased, while the magnitude of the performance differences between the training and validation sets varied across models. The training ROC-AUC of KNN remained close to 1.000 across different training sample sizes, whereas the validation ROC-AUC was approximately 0.85–0.90, indicating a relatively pronounced training–validation performance gap. The training ROC-AUCs of XGBoost, CatBoost, and LightGBM also remained high, while their validation ROC-AUCs generally remained within approximately 0.91–0.93; with increasing training sample size, the validation performance generally tended to stabilize. The differences between the training and validation ROC-AUCs of LR and SVM were relatively small and gradually stabilized as the training sample size increased. For RF, the training ROC-AUC gradually decreased and stabilized with increasing training sample size, whereas the validation ROC-AUC remained relatively stable. Overall, none of the models showed a continuous decline in validation performance with increasing training sample size; however, the magnitude of the performance differences between the training and validation sets varied across models.

**Figure 3:**
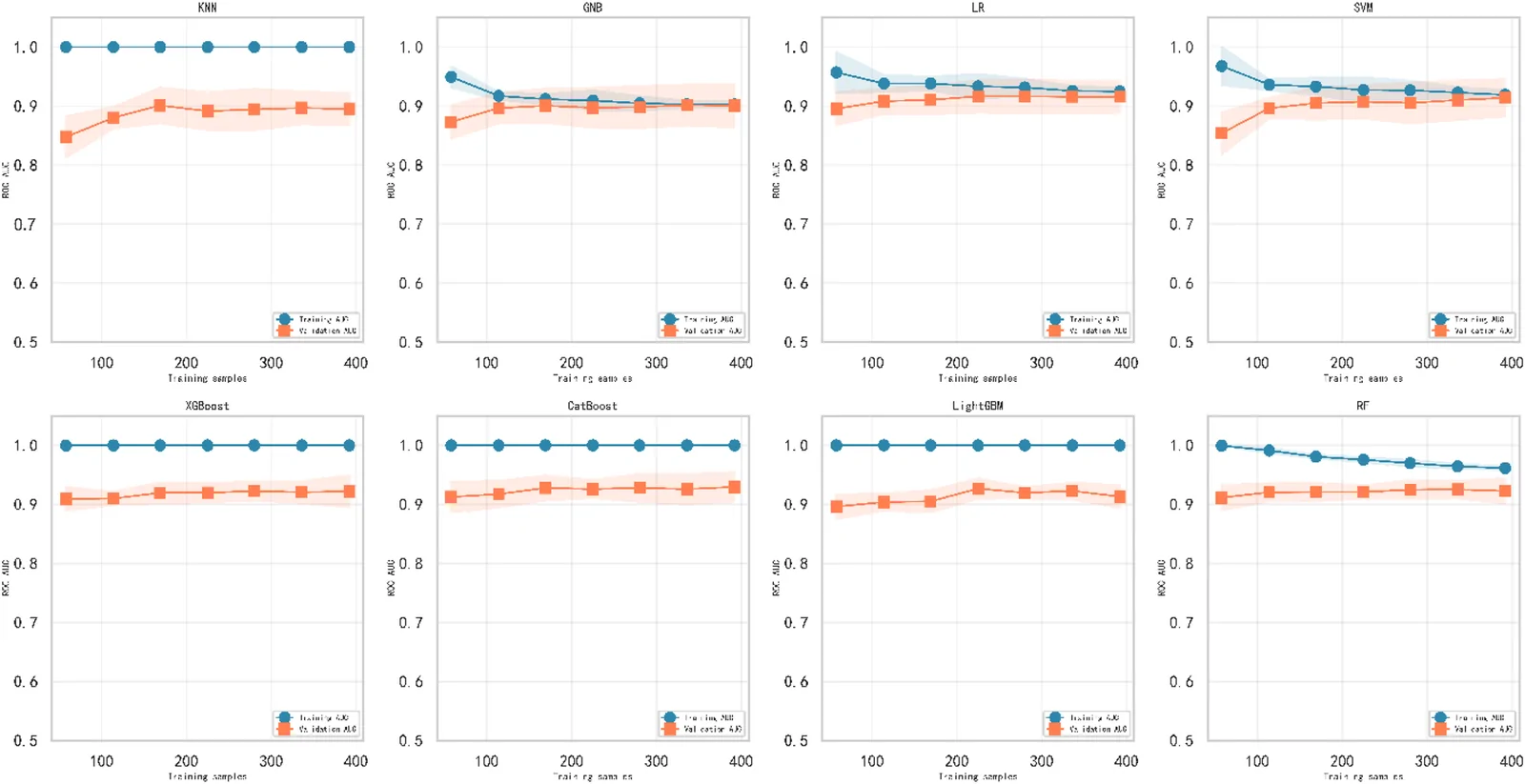
Learning curves of the eight models.

#### 3.3.3 Model performance comparison

In the test set, the performance of the eight models was further compared from multiple dimensions using ROC curves, PR curves, calibration curves, and decision curve analysis (DCA). The results are presented in Figures 4A–D and Tables 4–6.

**Figure 4:**
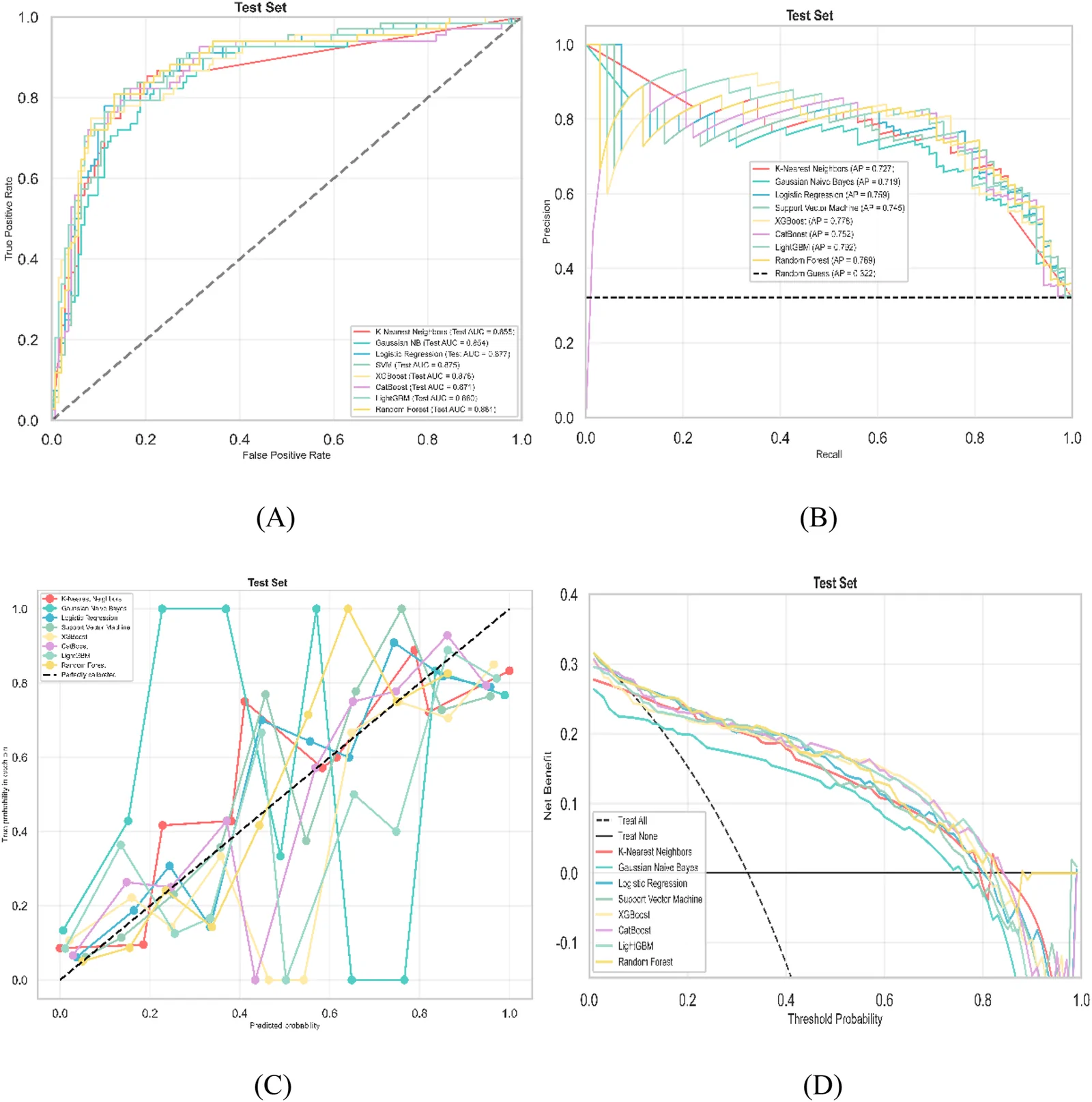
Performance assessment of eight machine learning models in the test set. **(A)** ROC curve; **(B)** PR curve; **(C)** calibration curve; **(D)** decision curve analysis.

**Table 4:** Comparison of *F*1 score differences between CatBoost and other models.

Model Comparison	*F*1-difference (95% CI)	*P* value	Bonferroni-adjusted *P* value
CatBoost vs. KNN	(−0.0034 to 0.1181)	0.062	0.434
CatBoost vs. GNB	(0.0253 to 0.1730)	0.006	0.042
CatBoost vs. LR	(−0.0173 to 0.1067)	0.133	0.931
CatBoost vs. SVM	(−0.0083 to 0.0940)	0.104	0.728
CatBoost vs. XGBoost	(−0.0197 to 0.0333)	0.535	1.000
CatBoost vs. LightGBM	(−0.0236 to 0.0496)	0.484	1.000
CatBoost vs. RF	(−0.0278 to 0.0372)	0.796	1.000

ROC curve analysis showed that all eight models demonstrated a certain degree of discriminative ability in the test set, with the curves generally above the random-classification reference line. The ROC-AUC values ranged from 0.854 to 0.881. The ROC-AUC values for RF, LightGBM, XGBoost, and LR were 0.881, 0.880, 0.878, and 0.877, respectively, while those for SVM, CatBoost, KNN, and GNB were 0.875, 0.871, 0.855, and 0.854, respectively (Figure 4A). PR curve analysis showed differences in precision–recall performance across models, with PR-AUC values ranging from 0.719 to 0.792. LightGBM, XGBoost, and RF showed relatively higher PR-AUC values of 0.792, 0.778, and 0.769, respectively (Figure 4B, Table 6). The calibration curves showed varying degrees of deviation between predicted probabilities and observed outcomes, with some models exhibiting fluctuations across different predicted-probability ranges (Figure 4C). These fluctuations may be partly attributed to the relatively limited test set size (*n* = 211), particularly the small number of patients within certain predicted-probability intervals, which may lead to unstable estimates of observed event rates. Additionally, differences in probability estimation among machine learning algorithms may contribute to variations in calibration performance. The Brier scores for RF and CatBoost were 0.122 and 0.123, respectively. The calibration slope and intercept were 1.077 and 0.089 for RF and 0.664 and −0.086 for CatBoost, respectively; the calibration metrics for the other models are presented in Table 6. Decision curve analysis showed differences in net benefit across models and threshold probabilities. Most models demonstrated higher net benefit than the “treat-all” and “treat-none” reference strategies over certain threshold ranges, while the curves showed a degree of overlap and intersection across a relatively broad range of thresholds (Figure 4D). To further compare performance differences between models, bootstrap difference testing was performed using CatBoost as the reference model, with Bonferroni correction applied for multiple comparisons. The *F*1 score comparisons are presented in Table 4. After Bonferroni correction, the difference between CatBoost and GNB was statistically significant (*P* = 0.042), whereas the differences between CatBoost and the other models were not statistically significant (*P* > 0.05). The ROC-AUC comparisons are presented in Table 5, and none of the differences between CatBoost and the other seven models remained statistically significant after Bonferroni correction (*P* > 0.05). Overall, the eight models demonstrated predictive capability in the test set, although their performance varied across different evaluation dimensions. RF showed relatively favorable performance in terms of ROC-AUC and Brier score, LightGBM had the highest PR-AUC, and CatBoost showed relatively high *F*1 score and accuracy. Given the absence of consistent statistically significant differences between models, no single model was designated as the uniquely optimal model based on a single evaluation metric or the performance of a single test set. Instead, the temporal generalizability of all models was further evaluated through temporal validation.

**Table 5:** Comparison of ROC-AUC differences between CatBoost and other models.

Model Comparison	ROC-AUC difference (95% CI)	*P* value	Bonferroni-adjusted *P* value
CatBoost vs. KNN	(−0.0221 to 0.0564)	0.441	1.000
CatBoost vs. GNB	(−0.0154 to 0.0507)	0.283	1.000
CatBoost vs. LR	(−0.0311 to 0.0181)	0.624	1.000
CatBoost vs. SVM	(−0.0324 to 0.0248)	0.824	1.000
CatBoost vs. XGBoost	(−0.0293 to 0.0146)	0.587	1.000
CatBoost vs. LightGBM	(−0.0346 to 0.0149)	0.467	1.000
CatBoost vs. RF	(−0.0247 to 0.0036)	0.156	1.000

**Table 6:** Probabilistic prediction and calibration performance of the eight models.

Model	PR-AUC	Brier	Calibration slope	Calibration intercept
KNN	0.727 (0.6074 to 0.8352)	0.138 (0.1023 to 0.1727)	0.110 (0.0801 to 0.1548)	−0.060 (−0.4470 to 0.3620)
GNB	0.719 (0.6014 to 0.8273)	0.173 (0.1287 to 0.2212)	0.151 (0.1066 to 0.2293)	−0.325 (−0.7250 to 0.1591)
LR	0.759 (0.6427 to 0.8633)	0.127 (0.0965 to 0.1606)	0.776 (0.5718 to 1.0891)	**−0.025** **(−0.4496 to 0.4387)**
SVM	0.745 (0.6298 to 0.8501)	0.130 (0.1011 to 0.1625)	0.855 (0.6188 to 1.2216)	−0.063 (−0.4935 to 0.4515)
XGBoost	0.778 (0.6659 to 0.8777)	0.131 (0.0946 to 0.1719)	0.509 (0.3946 to 0.6560)	−0.184 (−0.5928 to 0.1953)
CatBoost	0.752 (0.6373 to 0.8668)	0.123 (0.0903 to 0.1592)	0.664 (0.5066 to 0.8846)	−0.086 (−0.5039 to 0.3317)
LightGBM	**0.792** **(0.6827 to 0.8812)**	0.130 (0.0933 to 0.1707)	0.436 (0.3378 to 0.5730)	−0.203 (−0.6142 to 0.1749)
RF	0.769 (0.6562 to 0.8723)	**0.122** **(0.0954 to 0.1505)**	**1.077** **(0.8239 to 1.4564)**	0.089 (−0.3317 to 0.5333)

Note: Bold values indicate the best point estimates for each metric and their corresponding intervals: the highest PR-AUC, the lowest Brier score, the calibration slope closest to 1, and the calibration intercept closest to 0.

### 3.4 Temporal validation and bootstrap optimism correction

To further evaluate the temporal generalizability of the models and optimism in training-set performance, cases from 2023 to 2024 were used as the temporal training set, while cases from 2025 to 2026 were used as the temporal test set. In the temporal test set, the Accuracy, *F*1 score, ROC-AUC, PR-AUC, and Brier score ranged from 0.825–0.925, 0.759–0.900, 0.922–0.970, 0.873–0.956, and 0.073–0.134, respectively (Table 7). The Accuracy and *F*1 score of RF were 0.925 and 0.900, respectively, whereas CatBoost achieved an ROC-AUC of 0.970, a PR-AUC of 0.956, and a Brier score of 0.076. Bootstrap optimism correction was further performed within the 2023–2024 temporal training set, with the results presented in Supplementary Table S3. KNN showed the largest mean optimism in *F*1 score (0.113), whereas the mean optimism in AUC, *F*1 score, and Accuracy for RF was 0.020, 0.036, and 0.021, respectively. After optimism correction, the AUC, *F*1 score, and Accuracy of RF were 0.916, 0.763, and 0.862, respectively, compared with 0.969, 0.883, and 0.928 for CatBoost. Considering the overall results, RF and CatBoost were retained as complementary candidate models for subsequent model interpretation and risk prediction tool development. RF achieved the highest ROC-AUC (0.881) and the lowest Brier score (0.122) in the internal test set, as well as the highest Accuracy (0.925) and *F*1 score (0.900) in temporal validation. In contrast, CatBoost achieved the highest ROC-AUC (0.970) and PR-AUC (0.956) in temporal validation and maintained favorable performance after bootstrap optimism correction. Thus, their selection was based on complementary strengths across multiple evaluation dimensions rather than superiority in any single metric.

**Table 7:** Predictive performance of the eight models in the temporal test set.

Model	Accuracy	Precision	Recall	*F*1 score	ROC-AUC	PR-AUC	Brier
KNN	0.837 (0.750–0.912)	0.800 (0.647–0.933)	0.774 (0.625–0.912)	0.787 (0.656–0.886)	0.922 (0.856–0.971)	0.885 (0.778–0.963)	0.104 (0.068–0.145)
GNB	0.825 (0.738–0.900)	0.815 (0.654–0.952)	0.710 (0.540–0.867)	0.759 (0.618–0.868)	0.926 (0.861–0.974)	0.873 (0.759–0.962)	0.134 (0.078–0.199)
LR	0.875 (0.800–0.938)	0.818 (0.677–0.941)	0.871 (0.750–0.971)	0.844 (0.735–0.930)	0.956 (0.910–0.987)	0.931 (0.852–0.983)	0.097 (0.073–0.124)
SVM	0.900 (0.825–0.963)	0.871 (0.750–0.970)	0.871 (0.742–0.970)	0.871 (0.774–0.949)	0.961 (0.920–0.991)	0.936 (0.857–0.988)	0.100 (0.075–0.128)
XGBoost	0.913 (0.838–0.963)	0.875 (0.744–0.972)	0.903 (0.792–1.000)	0.889 (0.793–0.958)	0.968 (0.930–0.994)	0.955 (0.902–0.991)	**0.073** **(0.038–0.116)**
CatBoost	0.863 (0.787–0.925)	0.813 (0.667–0.935)	0.839 (0.700–0.962)	0.825 (0.714–0.912)	**0.970** **(0.936–0.993)**	**0.956** **(0.902–0.991)**	0.076 (0.046–0.110)
LightGBM	0.913 (0.850–0.975)	0.853 (0.724–0.968)	**0.936** **(0.840–1.000)**	0.892 (0.800–0.963)	0.965 (0.926–0.992)	0.949 (0.888–0.988)	0.080 (0.041–0.126)
RF	**0.925** **(0.863–0.975)**	**0.931** **(0.818–1.000)**	0.871 (0.742–0.970)	**0.900** **(0.809–0.969)**	0.965 (0.926–0.993)	0.943 (0.868–0.991)	0.090 (0.065–0.118)

Note: Bold values indicate the best results for each metric in the temporal validation set, with the corresponding intervals also bolded. Higher values indicate better performance for Accuracy, Precision, Recall, F1 score, ROC—AUC, and PR—AUC, whereas a lower Brier score indicates better probabilistic prediction performance.

### 3.5 Sensitivity analysis

To assess the impact of potential temporal variables on model performance, eight models were rebuilt after excluding fever duration and peak temperature and validated on the same 30% test set (Supplementary Table S4). Following exclusion, model Accuracy, *F*1 score and ROC-AUC ranged from 0.787–0.858, 0.622–0.773 and 0.852–0.883, respectively, with overall performance comparable to the primary analysis. XGBoost and LightGBM obtained Accuracy of 0.858 and *F*1 score of 0.773; RF had an Accuracy of 0.858, *F*1 score of 0.769 and ROC-AUC of 0.881. The results demonstrate that removing fever duration and peak temperature exerted no marked effect on model predictive performance, supporting the robustness of our primary findings.

### 3.6 SHAP-based model interpretation

To further interpret the predictive mechanisms of the Random Forest (RF) and CatBoost models, SHAP analysis was performed to assess the contribution of individual features to model predictions. The two models showed some differences in the ranking of the nine predictors, but demonstrated substantial consistency in identifying the core predictive features. In the RF model, LDH and pulmonary consolidation had the highest mean absolute SHAP values, followed by D-dimer, FDP, and CRP, whereas pulmonary consolidation and LDH showed the greatest contributions in the CatBoost model, followed by FDP, CRP, and D-dimer (Figures 5A,C). Both models identified pulmonary consolidation and LDH as the most important predictive features, indicating their relatively consistent contributions to PB risk prediction. The SHAP beeswarm plots further showed that the presence of pulmonary consolidation was predominantly associated with positive SHAP values, suggesting an overall contribution toward a higher predicted probability of PB. Higher levels of LDH, FDP, CRP, and D-dimer also generally showed positive SHAP contributions in both models, whereas lower values were more frequently distributed in the negative SHAP direction (Figures 5B,D). Fever duration, peak temperature, and WBC showed relatively smaller overall contributions but still exhibited some degree of inter-individual heterogeneity, while ALB had a relatively limited overall contribution in both models. Overall, although the RF and CatBoost models differed somewhat in their feature importance rankings, they showed substantial consistency in identifying pulmonary consolidation, LDH, and coagulation- and inflammation-related indicators as important contributors to model predictions, supporting the interpretability of the two models.

**Figure 5:**
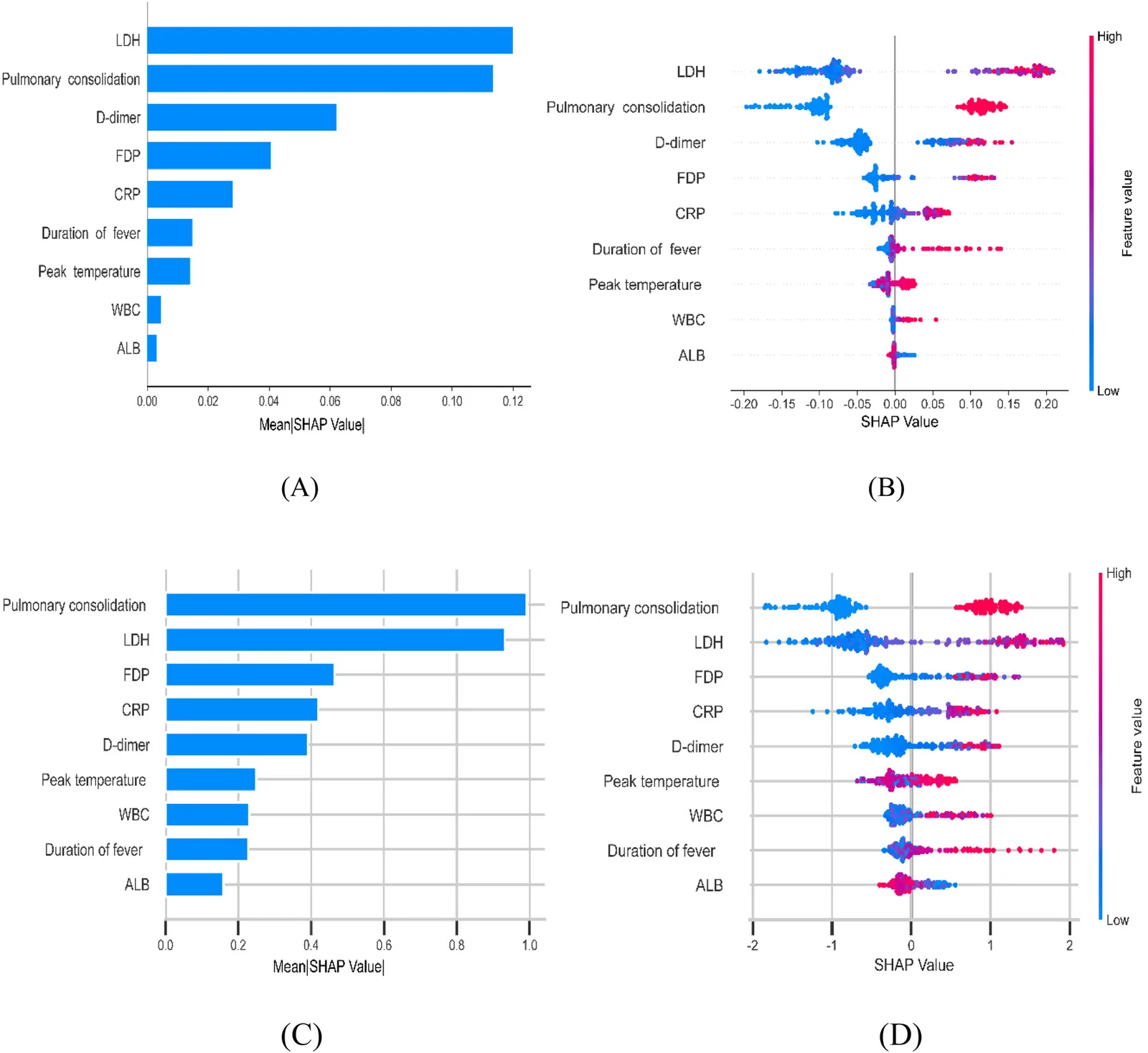
SHAP-based interpretation of the random forest and CatBoost models. **(A)** SHAP feature importance bar plot for the Random Forest model; **(B)** SHAP summary beeswarm plot for the Random Forest model; **(C)** SHAP feature importance bar plot for the CatBoost model; **(D)** SHAP summary beeswarm plot for the CatBoost model.

### 3.7 Development of RF- and CatBoost-based risk prediction calculators

Based on the nine predictors ultimately selected through the two-stage Lasso–Boruta feature selection procedure, two model-specific risk prediction calculators were developed and integrated into a single web-based application using the final Random Forest (RF) and CatBoost models, respectively. Both calculators incorporated the same nine predictors. After users entered the relevant clinical and laboratory characteristics of an individual patient, each calculator applied the corresponding final RF or CatBoost model to estimate the predicted probability of PB.

The RF risk calculator provided the predicted probability of PB, the corresponding RF decision threshold, and the resulting risk classification. The CatBoost risk calculator used the same input variables and prediction workflow but independently applied the final CatBoost model and its corresponding model-specific decision threshold.

In addition, to further facilitate individual-level model interpretability, both calculators incorporated SHAP-based analysis of individual feature contributions. The SHAP visualizations demonstrated the direction and relative magnitude of each predictor's contribution to the predicted risk, allowing the predicted probability to be interpreted in the context of the patient's specific clinical and laboratory characteristics. Overall, the two risk calculators established an integrated prediction workflow encompassing patient data input, PB risk probability estimation, model-specific threshold-based classification, and individual-level feature contribution interpretation, and may serve as research-oriented tools for PB risk assessment and model interpretation. Both calculators are publicly accessible through a single web application at https://pb-risk-tool.streamlit.app/. The interfaces of the two risk calculators are presented in Figures 6, 7.

**Figure 6:**
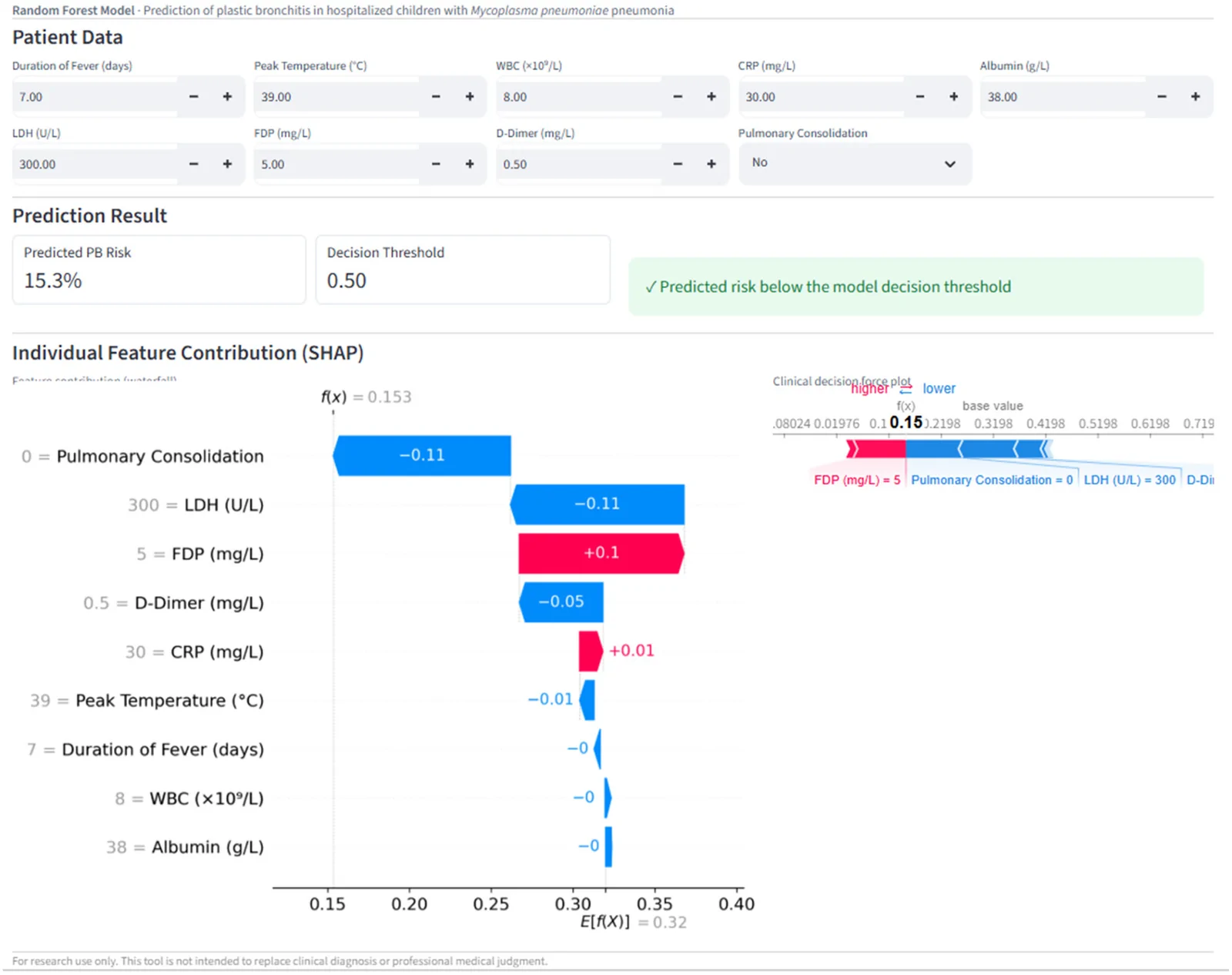
RF model risk calculator interface. The RF-based risk prediction calculator is publicly accessible at https://pb-risk-tool.streamlit.app/ (select “Random Forest”).

**Figure 7:**
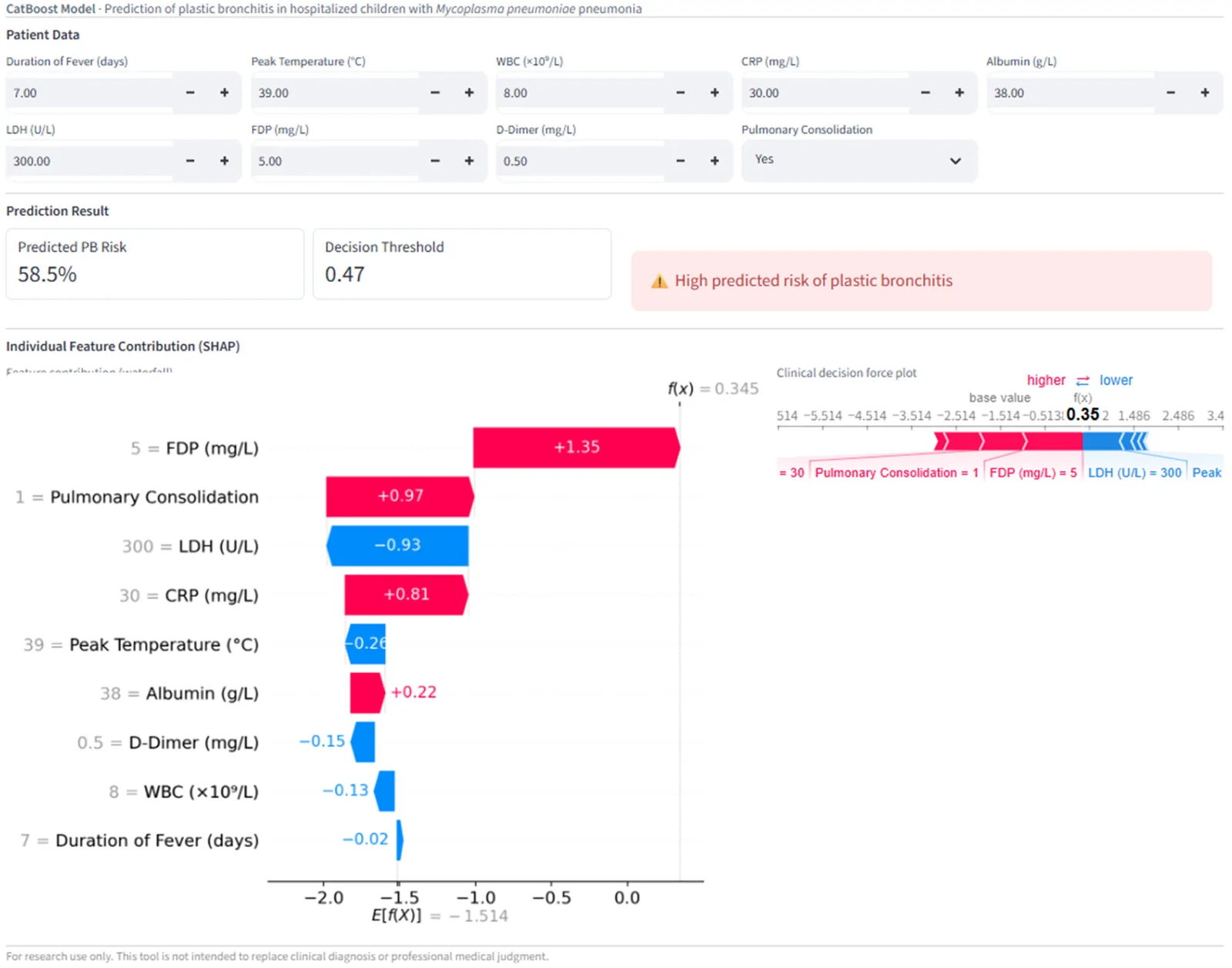
Catboost model risk calculator interface. The CatBoost-based risk prediction calculator is publicly accessible at https://pb-risk-tool.streamlit.app/ (select “CatBoost”).

## 4 Discussion

In recent years, with the deepening understanding of the clinical characteristics of MPP in children, a large body of research has confirmed that, compared with pneumonia caused by other pathogens, MPP in children is more prone to airway mucus plugging and cast formation, which may subsequently induce plastic bronchitis (18, 19). Therefore, early identification of PB complicating MPP remains a clinical challenge that needs to be addressed. Clinical guidelines (15) and related studies (20) have jointly suggested that early bronchoscopic intervention in MPP children with PB can effectively shorten the disease course and reduce the risk of long-term complications and sequelae. However, current clinical assessment mainly relies on physicians' experience and bronchoscopic findings, lacking objective and quantitative risk evaluation tools. The emergence of machine learning in disease prediction provides new perspectives for solving such problems, as ML models are better capable of capturing nonlinear relationships and interactions within data, thereby improving prediction accuracy and robustness (21). Therefore, this study utilized real-world data from 702 hospitalized children with MPP to develop and compare eight machine learning models for predicting the risk of PB. The results showed that children with PB exhibited differences in several clinical and laboratory indicators, including fever duration, peak temperature, CRP, LDH, and D-dimer, compared with those without PB. After two-stage Lasso–Boruta feature selection, all machine learning models constructed using the selected predictors demonstrated certain predictive capability, with ensemble tree-based models showing relatively better performance in some evaluation metrics. Considering the results from the independent test set, multidimensional model evaluation, temporal validation, and bootstrap optimism correction, RF and CatBoost were ultimately retained as candidate models for subsequent model interpretation and risk prediction tool development.

Given the “black-box” nature of machine learning models, SHAP (Shapley Additive Explanations) analysis was further performed to interpret the final retained RF and CatBoost candidate models at both the global and individual levels. Based on the nine PB-related predictors identified through the two-stage Lasso–Boruta feature selection approach, SHAP analysis demonstrated the contribution direction and relative importance of each variable to model predictions. Overall, pulmonary consolidation, LDH, FDP, CRP, D-dimer, peak temperature, WBC, fever duration, and ALB showed relatively high contributions in the two models, suggesting that these variables may play important roles in identifying PB risk among children with MPP. Consistent with the findings of Chu et al. (22), pulmonary consolidation was identified as an important predictor of PB in our study. This may be attributed to the enhanced inflammatory response associated with MPP complicated by PB, resulting in severe inflammatory infiltration of the bronchial wall and surrounding pulmonary parenchyma. Abundant fibrin and inflammatory exudates may accumulate within alveolar spaces. When inflammatory exudates extend into the bronchial lumen, fibrin-rich materials may combine with mucus, necrotic epithelial cells, and inflammatory cells, forming airway casts that are difficult to clear within a short period (22–26). LDH reflects the degree of pulmonary inflammation activation and tissue injury and has been associated with disease severity in MPP. Previous studies have reported elevated LDH levels as an independent risk factor for PB in children with MPP (27–30), which is consistent with our SHAP findings. Increased LDH may indicate enhanced pulmonary inflammatory activity and promote inflammatory cell infiltration into bronchial and peribronchiolar regions, thereby increasing the risk of airway obstruction. In the present study, increased FDP and D-dimer levels contributed to higher predicted PB risk. FDP and D-dimer represent activation of the coagulation–fibrinolysis system. MP infection can induce excessive inflammatory responses, promote local microthrombus formation, cause endothelial injury, and stimulate airway gland secretion, ultimately facilitating mucus and fibrin accumulation and cast formation (31–33). CRP and WBC, as inflammatory markers, also showed substantial contributions in the SHAP analyses of both models. Elevated CRP reflects systemic inflammatory activation and may promote complement activation and neutrophil recruitment. Activated inflammatory cells release proteolytic enzymes and reactive oxygen species, resulting in airway epithelial injury, impaired ciliary function, and reduced mucus clearance. The combination of inflammatory exudates and viscous airway secretions may contribute to cast formation (34). Peak temperature and fever duration also showed considerable contributions in model interpretation. Persistent fever may indicate sustained inflammatory activation, resulting in prolonged exposure of airway mucosa to inflammatory mediators, increased mucus production, impaired mucociliary clearance, and consequently elevated PB risk (24, 35). ALB showed relatively lower SHAP importance but was lower in the PB group and demonstrated a protective contribution. Hypoalbuminemia is frequently observed in severe infections, and reduced ALB levels in children with MPP may reflect persistent systemic inflammation and disease severity. Therefore, declining ALB levels may serve as a potential indicator for evaluating PB risk and disease progression (36). Overall, SHAP analysis provided further insight into the clinical characteristics contributing to machine-learning predictions, suggesting that enhanced inflammatory responses, coagulation–fibrinolysis activation, and pulmonary injury-related indicators collectively contribute to PB risk in children with MPP.

The present study systematically compared eight machine learning algorithms within a unified data framework. In the independent test cohort, the ROC-AUC values of all models ranged from 0.854 to 0.881, with relatively small differences among models. Bootstrap comparison using CatBoost as the reference model showed that none of the differences in ROC-AUC between CatBoost and the other models remained statistically significant after Bonferroni correction, while significant differences in *F*1 score were observed only between CatBoost and GNB. Both RF and CatBoost achieved an Accuracy of 0.853. The Precision, Recall, *F*1 score, and ROC-AUC of RF were 0.804, 0.721, 0.759, and 0.881, respectively, whereas those of CatBoost were 0.793, 0.736, 0.762, and 0.871, respectively. The two models demonstrated comparable overall performance but showed complementary characteristics across different evaluation dimensions. Therefore, this study did not determine a single “best” model based on any individual metric. Compared with previous studies, the XGBoost model developed by Wang et al. (13) achieved an AUC, sensitivity, specificity, and *F*1 score of 0.905, 0.812, 0.852, and 0.776, respectively, in the test cohort. The decision tree model developed by Li et al. (37) showed a sensitivity and specificity of 0.884 and 0.727, respectively. The CT radiomics-based model developed by Xu et al. (14) achieved an AUC, sensitivity, and specificity of 0.770, 0.720, and 0.760, respectively, in the test cohort. Zhao et al. (26) developed a nomogram based on Lasso combined with logistic regression, with AUC values of 0.813 in the development cohort and 0.895 in the subsequent temporal validation cohort. Although the performance of models across these studies fell within different ranges, direct comparisons should be interpreted cautiously because variations in study populations, PB prevalence, predictor variables, classification thresholds, and validation strategies may substantially influence model performance. Therefore, these results are more suitable for descriptive positioning rather than determining whether one model has universal superiority. Beyond discrimination performance, this study further evaluated PR-AUC, Brier scores, calibration, and decision curves. The PR-AUC values of the eight models ranged from 0.719 to 0.792. The Brier scores of RF and CatBoost were 0.122 and 0.123, respectively, which were numerically comparable to the Brier score of 0.124 reported for the XGBoost model developed by Wang et al. (13). The calibration slope and intercept of RF were 1.077 and 0.089, respectively, whereas those of CatBoost were 0.664 and −0.086. These findings indicate that although the two models showed similar discrimination ability and overall prediction error, their probability calibration characteristics differed. Therefore, the evaluation of clinical prediction models should not rely solely on AUC or a single classification metric, but should incorporate multiple dimensions, including discrimination, calibration, prediction error, and clinical net benefit. Temporal validation and bootstrap optimism correction further supported these findings. In the temporal test cohort from 2025 to 2026, RF achieved an Accuracy and *F*1 score of 0.925 and 0.900, respectively, whereas CatBoost achieved a ROC-AUC, PR-AUC, and Brier score of 0.970, 0.956, and 0.076, respectively. After bootstrap optimism correction, both models maintained relatively stable performance. Considering that missed identification of PB may delay subsequent bronchoscopy evaluation and intervention, sensitivity (Recall) was considered clinically important in interpreting model performance. However, optimizing sensitivity (Recall) alone may increase false-positive predictions; therefore, *F*1 score was retained as the primary classification performance criterion for model development and comparison, while Recall, Precision, Specificity, and other evaluation metrics were considered comprehensively. Based on the results from the independent test cohort, temporal validation, and multidimensional performance assessment, RF and CatBoost were ultimately retained as two candidate models and were further subjected to SHAP interpretation and web-based risk prediction tool development. Since the temporal validation cohort was derived from the same center, the external generalizability of these models requires further evaluation in independent multicenter prospective studies.

This study has several limitations. First, this was a single-center retrospective study, and all data were obtained from the Affiliated Hospital of Yan'an University in Yan'an, Shaanxi, China. Since the study population was restricted to hospitalized children with MPP who underwent bronchoscopy due to clinical indications, the cohort had potential selection bias, and the observed PB proportion should not be interpreted as the overall incidence among unselected children with MPP. In addition, formal stratification according to disease severity was not performed. Therefore, the applicability of the model to unselected MPP populations and patients with different disease severities remains to be further evaluated. Moreover, because calibration performance and decision-curve net benefit may vary with disease prevalence and case mix, these results should not be directly extrapolated to unselected MPP populations with different PB prevalence. Second, all laboratory indicators were obtained from a single measurement within 24 h after admission, representing cross-sectional data that could not capture dynamic changes during disease progression. Temporal variations in biomarkers may provide additional predictive information that was not evaluated in this study. Third, due to the retrospective design, the exact timing of bronchoscopy and PB confirmation was not systematically recorded. Although admission was defined as the prediction time origin and all predictors were collected before PB confirmation, the potential time benefit of model-assisted risk identification for subsequent diagnosis and intervention could not be quantified. Future prospective studies should record the timing of model assessment, clinical decisions, and bronchoscopy intervention to further evaluate clinical utility. Finally, although 5-fold cross-validation was used for hyperparameter optimization, and temporal validation and bootstrap optimism correction were performed, independent external validation was not conducted. All patients were recruited from a single center in Yan'an, and racial/ethnic background was not systematically recorded; therefore, the performance of the models across different geographic regions, racial/ethnic populations, and age groups could not be evaluated. Moreover, some potentially relevant biomarkers, such as cytokine profiles and ferritin, were not included. Future multicenter prospective studies are needed to externally validate the models and evaluate the clinical applicability of the developed web-based risk prediction calculator.

## 5 Conclusions

1. Through two-stage Lasso–Boruta feature selection, nine PB-related predictors were identified, including fever duration, peak temperature, WBC, CRP, ALB, LDH, FDP, D-dimer, and pulmonary consolidation.
2. Based on these predictors, eight machine learning models were developed and evaluated. RF and CatBoost showed stable and complementary predictive performance, and combined with SHAP interpretation and web-based risk prediction tools, may provide an interpretable approach for early PB risk identification in children with MPP. Further multicenter external validation is required.

## Data Availability

The raw data supporting the conclusions of this article will be made available by the authors, without undue reservation.
