# Supplementary material for: Machine learning models for predicting plastic bronchitis in hospitalized children with Mycoplasma pneumoniae pneumonia undergoing clinically indicated bronchoscopy: a single-center retrospective study
Source: Front Pediatr. 2026 Oct 30;14:1983032. doi: 10.3389/fped.2026.1983032 (PMC13759768; doi:10.3389/fped.2026.1983032)
Supplement: Supplementary file 1 [file Datasheet1.pdf]

Supplementary Material

1 Supplementary Figures and Tables

Table S1. Hyperparameter Search Spaces and Optimal Hyperparameters for the Eight Machine Learning Models

Model Name	Parameter Range	Optimal Parameters
KNN	{'n_neighbors': [1, 3, 5, 7, 9, 11, 13, 15, 20, 30], 'weights': ['uniform', 'distance'], 'metric': ['euclidean', 'manhattan', 'minkowski']}	{'metric': 'euclidean', 'n_neighbors': 5, 'weights': 'distance'}
GNB	{'var_smoothing': [1e-09, 1e-08, 1e-07, 1e-06, 1e-05, 0.0001, 0.001]}	{'var_smoothing': 1e-09}
LR	{'C': [0.001, 0.01, 0.1, 1, 10, 100], 'penalty': ['l1', 'l2'], 'solver': ['liblinear', 'saga'], 'max_iter': [1000, 2000]}	{'C': 1, 'max_iter': 1000, 'penalty': 'l1', 'solver': 'liblinear'}
SVM	{'C': [0.1, 1, 10, 100], 'kernel': ['linear', 'rbf', 'poly'], 'gamma': ['scale', 'auto', 0.001, 0.01, 0.1, 1]}	{'C': 10, 'gamma': 'scale', 'kernel': 'linear'}
XGBoost	{'n_estimators': [100, 200, 300], 'max_depth': [3, 4, 5, 6], 'learning_rate': [0.01, 0.1, 0.2], 'subsample': [0.8, 0.9, 1.0], 'colsample_bytree': [0.8, 0.9, 1.0]}	{'colsample_bytree': 0.9, 'learning_rate': 0.1, 'max_depth': 6, 'n_estimators': 100, 'subsample': 0.8}
CatBoost	{'iterations': [100, 200, 300], 'depth': [4, 6, 8], 'learning_rate': [0.01, 0.1, 0.2], 'l2_leaf_reg': [1, 3, 5, 7, 9]}	{'depth': 8, 'iterations': 100, 'l2_leaf_reg': 1, 'learning_rate': 0.1}
LightGBM	{'n_estimators': [100, 200, 300], 'max_depth': [3, 4, 5, 6], 'learning_rate': [0.01, 0.1, 0.2], 'num_leaves': [31, 50, 100], 'min_child_samples': [10, 20, 30]}	{'num_leaves': 100, 'n_estimators': 100, 'min_child_samples': 10, 'max_depth': 5, 'learning_rate': 0.1}
RF	{'n_estimators': [100, 200, 300, 500], 'max_depth': [None, 3, 5, 7, 10], 'min_samples_split': [2, 5, 10], 'min_samples_leaf': [1, 2, 4], 'max_features': ['sqrt', 'log2', None], 'bootstrap': [True, False]}	{'bootstrap': False, 'max_depth': 3, 'max_features': 'sqrt', 'min_samples_leaf': 2, 'min_samples_split': 2, 'n_estimators': 500}

Table S2. Five-fold Cross-validation Performance of the Eight Machine Learning Models

Model	Accuracy	Precision	Recall	F1 score	AUC
KNN	0.876 ± 0.051 (0.816-0.929)	0.842 ± 0.102 (0.697-0.963)	0.757 ± 0.089 (0.613-0.844)	0.795 ± 0.085 (0.691-0.881)	0.883 ± 0.044 (0.828-0.931)
GNB	0.847 ± 0.042 (0.786-0.888)	0.841 ± 0.100 (0.667-0.917)	0.656 ± 0.093 (0.516-0.774)	0.732 ± 0.074 (0.653-0.814)	0.899 ± 0.046 (0.827-0.945)
LR	0.890 ± 0.040 (0.847-0.929)	0.857 ± 0.085 (0.722-0.929)	0.796 ± 0.096 (0.645-0.903)	0.821 ± 0.067 (0.727-0.889)	0.915 ± 0.044 (0.850-0.963)
SVM	0.890 ± 0.042 (0.837-0.929)	0.857 ± 0.088 (0.714-0.931)	0.796 ± 0.087 (0.677-0.903)	0.822 ± 0.067 (0.750-0.889)	0.913 ± 0.043 (0.849-0.962)
XGBoost	0.886 ± 0.038 (0.837-0.929)	0.843 ± 0.090 (0.714-0.962)	0.803 ± 0.099 (0.710-0.968)	0.818 ± 0.058 (0.758-0.896)	0.932 ± 0.030 (0.897-0.971)
CatBoost	0.888 ± 0.040	0.864 ± 0.093	0.783 ± 0.095	0.817 ± 0.065	0.936 ± 0.030

	(0.837-0.929)	(0.714-0.963)	(0.645-0.903)	(0.755-0.889)	(0.894-0.975)
LightGBM	0.878 ± 0.025<br>(0.847-0.908)	0.825 ± 0.057<br>(0.735-0.893)	0.790 ± 0.073<br>(0.710-0.903)	0.805 ± 0.041<br>(0.769-0.862)	0.921 ± 0.036<br>(0.877-0.967)
RF	0.884 ± 0.030<br>(0.837-0.918)	0.865 ± 0.079<br>(0.727-0.923)	0.765 ± 0.070<br>(0.677-0.871)	0.808 ± 0.047<br>(0.750-0.871)	0.931 ± 0.029<br>(0.896-0.968)

Note: Data are presented as mean ± standard deviation (SD), with the values in parentheses representing the minimum–maximum range (Range) of performance across the five cross-validation folds.

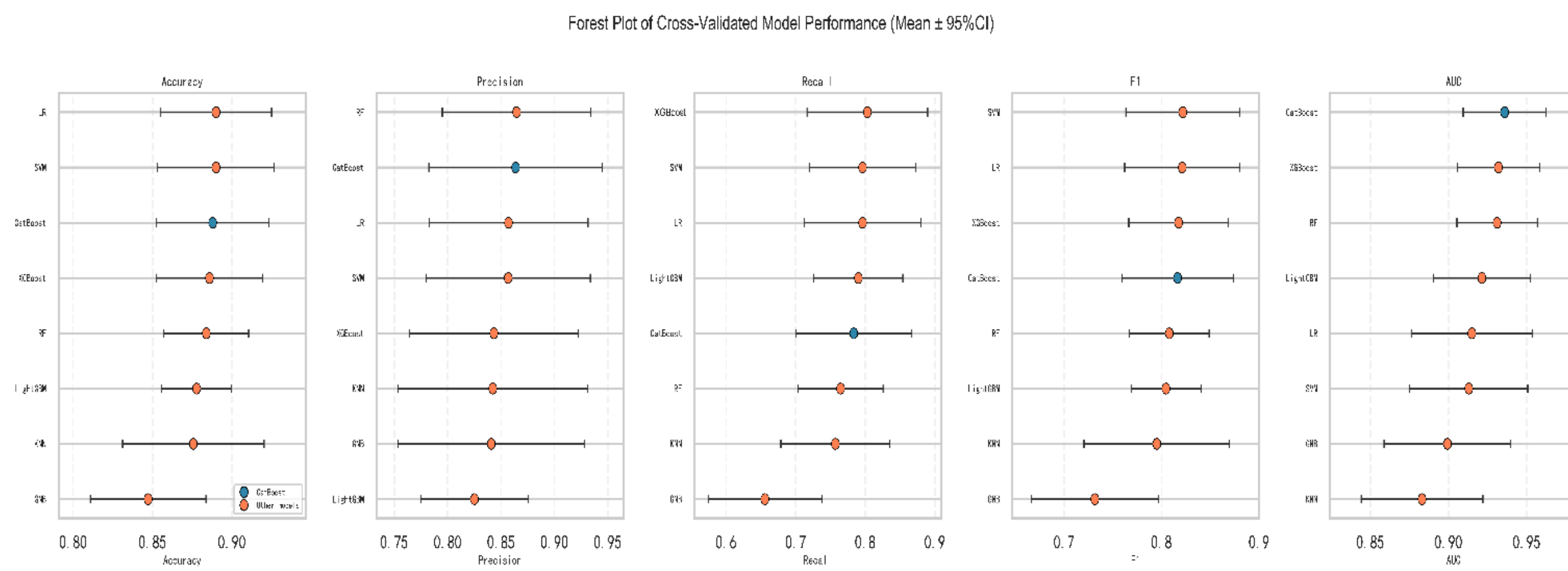

Figure S1. Forest Plot of Five-fold Cross-validation Performance of the Eight Machine Learning Models.

Note: The plots present the mean values and corresponding 95% confidence intervals (CIs) for Accuracy, Precision, Recall, F1 score, and ROC-AUC. Blue circles represent the CatBoost model, whereas orange circles represent the other seven machine learning models. Horizontal black error bars indicate the corresponding 95% CIs.

Table S3. Bootstrap optimism correction for the eight prediction models

Model	Metric	Apparent performance	Mean optimism	Optimism 95% CI	Optimism-corrected performance
KNN	AUC	1.000	0.042	0.027–0.059	0.958
	F1	1.000	0.113	0.084–0.142	0.887
	Accuracy	1.000	0.069	0.051–0.087	0.931
	Brier	0.000	0.054	0.043–0.067	0.054
GNB	AUC	0.883	0.003	−0.029–0.034	0.880
	F1	0.685	0.003	−0.054–0.056	0.681
	Accuracy	0.830	0.002	−0.026–0.031	0.827
	Brier	0.140	0.002	−0.023–0.027	0.142
LR	AUC	0.904	0.006	−0.021–0.031	0.898
	F1	0.775	0.012	−0.040–0.057	0.764
	Accuracy	0.867	0.007	−0.019–0.034	0.859
	Brier	0.109	0.005	−0.010–0.022	0.114
SVM	AUC	0.901	0.007	−0.022–0.035	0.894
	F1	0.756	0.013	−0.036–0.059	0.743
	Accuracy	0.857	0.008	−0.019–0.035	0.849
	Brier	0.114	0.006	−0.009–0.023	0.119
XGBoost	AUC	1.000	0.031	0.019–0.044	0.969

	F1	0.997	0.087	0.065–0.112	0.910
	Accuracy	0.998	0.054	0.040–0.069	0.945
	Brier	0.009	0.043	0.032–0.054	0.051
CatBoost	AUC	0.998	0.028	0.018–0.041	0.969
	F1	0.969	0.086	0.063–0.110	0.883
	Accuracy	0.981	0.052	0.039–0.066	0.928
	Brier	0.026	0.038	0.029–0.049	0.064
LightGBM	AUC	1.000	0.033	0.021–0.048	0.967
	F1	1.000	0.088	0.064–0.113	0.912
	Accuracy	1.000	0.054	0.040–0.069	0.946
	Brier	0.006	0.045	0.033–0.057	0.050
RF	AUC	0.936	0.020	−0.000–0.038	0.916
	F1	0.799	0.036	−0.004–0.073	0.763
	Accuracy	0.883	0.021	−0.003–0.042	0.862
	Brier	0.091	0.013	0.001–0.026	0.104

Table S4. Sensitivity analysis of the eight models after excluding fever duration and peak temperature

Model	Accuracy	Precision	Recall	F1 Score	AUC-ROC
XGBoost	**0.858 (0.810–0.905)**	0.797 (0.695–0.894)	**0.750 (0.644–0.850)**	**0.773 (0.684–0.848)**	0.882 (0.827–0.932)
LightGBM	**0.858 (0.810–0.905)**	0.797 (0.697–0.895)	**0.750 (0.645–0.852)**	**0.773 (0.686–0.848)**	**0.883 (0.830–0.932)**
RF	**0.858 (0.810–0.905)**	**0.806 (0.703–0.900)**	0.735 (0.627–0.838)	0.769 (0.682–0.846)	0.881 (0.822–0.933)
CatBoost	0.853 (0.806–0.900)	0.803 (0.699–0.900)	0.721 (0.613–0.824)	0.760 (0.672–0.837)	0.876 (0.820–0.929)
LR	0.839 (0.787–0.886)	0.793 (0.686–0.897)	0.676 (0.563–0.782)	0.730 (0.636–0.810)	0.875 (0.819–0.925)
SVM	0.820 (0.768–0.872)	0.742 (0.630–0.848)	0.676 (0.562–0.784)	0.708 (0.610–0.792)	0.876 (0.820–0.927)
KNN	0.810 (0.754–0.863)	0.741 (0.625–0.851)	0.632 (0.521–0.745)	0.683 (0.579–0.772)	0.877 (0.821–0.926)
GNB	0.787 (0.734–0.839)	0.725 (0.604–0.845)	0.544 (0.421–0.662)	0.622 (0.505–0.719)	0.852 (0.793–0.909)
